# Tiny Screens, Big Impact: Effects of Maternal Smartphone Use on Maternal and Infants' Physiological and Behavioral Stress and Interaction Dynamics

**DOI:** 10.1111/infa.70056

**Published:** 2025-12-06

**Authors:** Antonia Dinzinger, Elke Greif, Lydia Speyer, Ralf Arne Wittling, Karl Heinz Brisch, Beate Priewasser, Gabriela Markova

**Affiliations:** ^1^ Institute for Early Life Care Paracelsus Medical University Salzburg Austria; ^2^ Department of Pediatrics University Hospital Salzburg Paracelsus Medical University Salzburg Austria; ^3^ ZNF—Center for Neuroscience Research NPO Trier Germany; ^4^ Dr. von Hauner Children's Hospital Ludwig Maximilians University Munich Germany

## Abstract

Smartphones can absorb attention and abruptly interrupt social interactions, a dynamic particularly critical in early parent‐infant exchanges where infants rely on emotionally available caregivers for regulation. While previous research highlights the negative effects of parental media use on parenting and infant behavior, little is known about how maternal smartphone use affects both mothers' and infants' physiological and behavioral stress responses during early interactions. Consequently, we observed 67 mothers and their 6‐month‐old infants during an extended still‐face paradigm including five phases in total: (1) an interaction baseline, (2) a still‐face interruption, (3) a subsequent reunion, (4) a smartphone interruption, and (5) a subsequent reunion. The order of interruptions was randomized. Maternal and infant heart rates were continuously recorded via electrocardiogram. Infant protest and self‐regulatory behaviors, as well as maternal co‐regulatory behaviors, were coded from video recordings on a frame‐by‐frame basis. Infants showed significantly more protest behavior, higher cardiac arousal, and reduced parasympathetic activity during maternal smartphone use compared to baseline. During smartphone use, mothers reduced social engagement, accompanied by increased parasympathetic activity and decreased physiological arousal, which remained lower during reunions. We also found that dyadic physiological coupling emerged during still‐face and smartphone disruptions, whereas dyadic behavioral coupling was observed only during the baseline interaction. These findings highlight the disruptive effects of maternal smartphone use and raise important questions about the potential cumulative effects of repeated smartphone interruptions on early socioemotional development.

## 1 Introduction

During the 1st years of life, infants and young children rely on emotionally available caregivers to meet their physical and emotional needs (Perry et al. 2017). Sensitive parent‐child interactions are characterized by attentive parenting, whereby parents are attuned to their child’s communicative signals, interpret them accurately, and respond promptly and appropriately (Ainsworth et al. 1978). This sensitivity is especially critical in early infancy, when self‐regulatory capacities are still developing and children are dependent on external regulation (e.g., A. S. Morris et al. 2017). However, the increasing ubiquity of smartphones has introduced a significant source of distraction, as parents may shift attention away from their child to use these devices for communication, information seeking, or entertainment. Research indicates that increased digital engagement with a computer or smartphone is linked to reduced feelings of connectedness towards individuals in one's immediate physical presence (Nguyen et al. 2021; Venter 2019). McDaniel and Coyne (2016) coined the term “technoference” to describe such technology‐induced interruptions in social interactions. In addition, smartphone use often leads to states of deep absorption or immersion, during which users disengage from their surroundings for extended periods. These phenomena raise concerns about the impact of parental smartphone use on early parent‐infant interactions. Indeed, studies have shown that smartphone use by parents is associated with less sensitive parenting behaviors (e.g., Abels et al. 2018; Elias et al. 2021; Konrad, Hillmann, et al. 2021) and adverse effects on infant behavior (e.g., Elias et al. 2021; Lemish et al. 2020) and physiological arousal (Rozenblatt‐Perkal et al. 2022). Beyond immediate effects on parent‐child interactions, increased parental technology use has been linked to lower mother‐child attachment quality (Alvarez Gutierrez and Ventura 2021), poorer child self‐regulation and response inhibition, more behavioral problems (Carson and Kuzik 2021; Yang et al. 2023), and weaker vocabulary development (Corkin et al. 2021). Despite these findings, little is known about how parental smartphone use during early interactions influences both mothers' and infants' behavioral and autonomic stress responses. Furthermore, the way parenting behavior during and following smartphone use relates to infants' behavioral and cardiac stress responses remains underexplored. To address these gaps, the present study employed a multi‐method approach to investigate the impact of smartphone‐induced disruptions on early mother‐infant interactions.

A recent review concluded that smartphone‐related technoference is negatively associated with both parenting and child behavior (Komanchuk et al. 2023). Early laboratory work demonstrated that mothers who spontaneously used their phones during a structured play task engaged in significantly fewer verbal and non‐verbal interactions with their toddlers compared to mothers who refrained from phone use (Radesky et al. 2015). Parents who use their smartphones during interactions with their young children tend to be less sensitive and responsive (Kildare and Middlemiss 2017), because it is negatively associated with the likelihood, timeliness, and quality of their responses (Abels et al. 2018). Moreover, smartphone use has been found to be more disruptive compared to other distractions, such as paper‐and‐pencil tasks or other activities, resulting in reduced responsiveness and joint attention (Konrad, Hillmann, et al. 2021; Krapf‐Bar et al. 2022). Even in interactions with older children, parental smartphone use is linked to reduced responsiveness, fewer verbal interactions, and less joint attention both at home and in public settings (Lederer et al. 2022; Ochoa et al. 2021; vanden Abeele et al. 2020). In addition to compromising the quality of parental attention and sensitivity of responses, parents report reduced feelings of social connectedness with their children (Kushlev and Dunn 2019). Yet, it remains unclear whether smartphone use reduces parental responsiveness, or whether less sensitive parents are more likely to use their phones while engaged with their child.

Despite the negative association between smartphone‐related interruptions and parental behavior, evidence suggests that parents endeavor to re‐engage with their children after an interruption caused by a smartphone. For instance, mothers interacting with their 20‐ to 22‐month‐old toddlers showed reduced responsiveness and pedagogical behavior during smartphone use, while these behaviors tended to return to baseline levels once the smartphone interruption ends (Konrad, Hillmann, et al. 2021). Similarly, joint attention between mothers and their 11‐month‐old infants decreased during smartphone use but recovered after the episode of technoference (Krapf‐Bar et al. 2022). At the same time, many parents express a desire to change their smartphone use habits (McDaniel et al. 2023) and their re‐engagement attempts after smartphone use appear to vary, potentially influenced by the frequency of their daily smartphone use (Myruski et al. 2018). However, it remains unclear which parental behaviors during and after smartphone use can mitigate its impact on their interaction with the child. It is unclear whether it is sufficient for parents to re‐engage with their children after a smartphone interruption, or whether maintaining parental responsiveness during the actual use can buffer the negative effects of technoference or absorption. There is evidence showing that verbal interactions during media exposure can mitigate negative effects on long‐term language development in 6‐month‐old infants (Mendelsohn et al. 2010). While this study focused on media exposure rather than parental phone use, it raises the question of whether the quality of parental engagement during smartphone use might similarly influence child outcomes. Thus, the child's immediate experience may not only be shaped by the mere presence of smartphone use but also by how emotionally available the parent remains during that time.

At the same time, emerging evidence suggests that children also adjust their interactive behavior in response to parental smartphone use. They display more attention seeking behavior, express more negative affect, or show signs of withdrawal from any attempt to communicate with their parents (e.g., Alvarez Gutierrez and Ventura 2021; Elias et al. 2021; Gaudreau et al. 2022; Kildare and Middlemiss 2017; Myruski et al. 2018; Stockdale et al. 2020). These infant behaviors are comparable to those resulting from being completely ignored, such as during the still‐face. The still‐face paradigm (SFP) provides a well‐established framework for examining infants' behavioral and physiological responses to temporary disruptions in caregiver responsiveness. It reliably elicits increase in arousal and distress during the interruption phase, whereas the reunion phase following the interruption reveals infants' recovery and regulatory responses (Conradt and Ablow 2010; Mesman et al. 2009). The SFP thus serves as a benchmark for understanding the effects of parental smartphone use, which similarly reduces responsiveness. In fact, parents display still‐face behavior during smartphone use about 77% of the time (Konrad, Berger‐Hanke, et al. 2021), supporting comparability between the two contexts (see also Myruski et al. 2018; Stockdale et al. 2020). Unlike the classic SFP, smartphone use presents a visible external cause for parental unavailability, which may make the interruption less ambiguous but still disruptive to infants (e.g., Legerstee and Markova 2007).

Parental smartphone use during early interactions may not only affect interactive behavior but also elicit immediate physiological responses in both interactive partners (Mikić and Klein 2022). Parental heart rate (HR) and respiratory sinus arrhythmia (RSA) are closely linked to emotional availability and sensitivity (Connell et al. 2017; Joosen et al. 2013; Zhang et al. 2017). HR reflects general autonomic arousal, while RSA, an index of parasympathetic control, relates to attentional engagement and affect regulation (Gordan et al. 2015; Porges 2007). Studies using the SFP demonstrate that infants' HR increases from free‐play to the still‐face phase (e.g., Conradt and Ablow 2010; Gunning et al. 2013), while during the reunion phase infant HR tends to slightly decrease as they begin to recover (Moore and Calkins 2004; Mesman et al. 2009). Concurrently, infant RSA decreases during still‐face and partially recovers during reunion, while maternal RSA often increases during still‐face and decreases during reunion, reflecting regulatory efforts (Moore and Calkins 2004; Moore et al. 2009; Oppenheimer et al. 2013; Conradt and Ablow 2010). Together, these patterns suggest that arousal and parasympathetic regulation capture complementary aspects of dyadic physiological adaptation.

In fact, research on parent‐child physiological coordination found positive HR coupling during behavioral coordination, highlighting the alignment of behavioral and physiological dynamics (Feldman et al. 2011), and its persistence across maternal affective states (Field et al. 1989). Mother‐to‐infant HR coupling has been observed during vocalizations (McFarland et al. 2019) and even at rest (Moore et al. 2009), suggesting that it reflects both adaptive attunement and stress vulnerability (Waters et al. 2014). RSA coupling, in contrast, is highly context dependent. It serves a coregulatory function early in life, facilitating infants' distress recovery and emerging self‐regulation (Abney, daSilva, and Bertenthal 2021b; Feldman 2007, 2012; Stallworthy et al. 2024). Positive RSA coupling typically occurs in low‐stress contexts such as play, habituation, and problem‐solving tasks (Abney, daSilva, and Bertenthal 2021b; Feldman et al. 2011; Bornstein and Suess 2000; Wass et al. 2019), whereas negative coupling emerges during or after distress (Moore et al. 2009; Ostlund et al. 2017). Moment‐by‐moment analyses revealed that dyads flexibly shift in and out of RSA synchrony to meet contextual demands (Abney, daSilva, and Bertenthal 2021b; Feldman 2007; Mayo and Gordon 2020). Thus, negative feedback loops seem to support shared physiological states (Smith et al. 2022).

Parental smartphone use provides a real‐world example of how these processes may be disrupted. Studies show that infant HR increases during parental smartphone engagement, while mother‐child RSA coupling decreases, reflecting impaired parasympathetic co‐regulation (Porter et al. 2024; Rozenblatt‐Perkal et al. 2022; Stallworthy et al. 2024). Notably, HR reflects infants' immediate arousal response to parental distraction, whereas RSA captures the dyad's compromised regulatory coordination, highlighting the need to consider both measures when examining disruptions in early regulatory processes. However, little is known about how mothers adapt to infants' distress while using a smartphone. Such adaptions may involve changes in arousal, parasympathetic activity, and physiological‐behavioral coupling during and after the interruption. Moreover, individual differences, such as parental habitual smartphone use, may further shape these dynamics. Some research has linked higher maternal habitual smartphone use to reduced child positive affect (Myruski et al. 2017) and greater object orientation (Stockdale et al. 2020), while other studies found no association with children's emotional responses (Rozenblatt‐Perkal et al. 2022). It remains unclear whether habitual smartphone use modulates maternal behavior during and after smartphone interruptions, and how such changes affect infants' physiological and behavioral responses.

### 1.1 The Present Study

To address these gaps, in the present study we used a multi‐method approach combining behavioral and physiological measures to examine the effects of smartphone‐induced interruptions during early interactions. We have extended the SFP (Tronick et al. 1978) by adding a second interruption phase where mothers were instructed to solve a crossword puzzle on a smartphone. Based on the literature reviewed above, we formulated the following hypothesis.

First, we hypothesized that infants experience the smartphone perturbation as stressful, as evidenced by increased protest behavior, more self‐regulatory behavior, as well as higher cardiac arousal during the smartphone compared to the baseline phase.

Second, we hypothesized that mothers show reduced interactive behaviors during smartphone use compared to baseline, and that these behaviors increase following both smartphone and still‐face interruptions. Yet, we assumed that maternal daily smartphone use will be negatively associated with their engagement during and after the smartphone perturbation. We further expected lower cardiac arousal during both interruption phases and a recovery to baseline levels during the reunions following both smartphone and still‐face phases.

We hypothesized dynamic coupling between social engagement behaviors as well as cardiac activity of mothers and their infants, depending on the condition of the procedure.

## 2 Method

### 2.1 Participants

Overall, 67 mother‐child dyads were recruited at local playgroups, pediatricians' offices, baby stores, and by social media advertisements. Participation was voluntary and was monetarily compensated. Data from two dyads were excluded entirely because the experiment had to be terminated early due to excessive infant distress. This resulted in a sample of 65 white, middle class mothers (*M* = 32 years, SD = 3.96, range = 26–43) and their children (59% girls; *M* = 6 months, SD = 1.93, range = 3–10). All mothers were in a committed relationship. Only mothers over 18 years of age and children born at term and without any known neurological impairments were included. Specific phases from two dyads were excluded because mothers did not follow the instructions (e.g., talking during the still‐face). Additionally, four dyads did not complete all experimental phases but were included in the analyses for the phases they completed, and three mothers did not complete all questionnaires. All mothers gave written informed consent prior to the experiment. For further sample characteristics see Table 1.

**TABLE 1:** Sample characteristics.

Variable	Value
Infant age, months, *M* (SD)	6.11 (1.93)
Infant gender, *n* (%)	
Male	27 (41.5)
Female	38 (58.5)
Mother age, years, *M* (SD)	32.77 (3.96)
Marital status, *n* (%)	
In a committed relationship	62 (95.4)
Single mother	0 (0)
Missing	3 (4.6)
Employment (multiple choice), *n* (%)	
Maternal leave	60 (9.3)
Full time employment	0 (0)
Part time employment	2 (3.1)
Housewife	0 (0)
Training/studies	2 (3.1)
Unemployed	0 (0)
Marginal employment	2 (3.1)
Self employment	4 (6.2)
Missing	3 (4.6)
Education level, *n* (%)	
Professional training	6 (9.2)
General qualification for university entrance	8 (12.3)
University degree	46 (70.8)
Other	2 (3.1)
Missing	3 (4.6)
Nationality, *n* (%)	
Austria	53 (81.5)
Germany	5 (7.7)
Other	4 (6.2)
Missing	3 (4.6)
Yearly household net income, *n* (%)	
< 32,000 €	5 (7.7)
32,000–48,000 €	28 (43.1)
48,000–67,000 €	21 (32.3)
> 67,000 €	8 (12.3)
Missing	3 (4.6)
Daily smartphone use, *n* (%)	
0,5–1 h	17 (26.2)
1–3 h	34 (52.3)
3–5 h	7 (10.8)
> 5 h	4 (6.2)
Missing	3 (4.6)

### 2.2 Procedure

**FIGURE 1:**
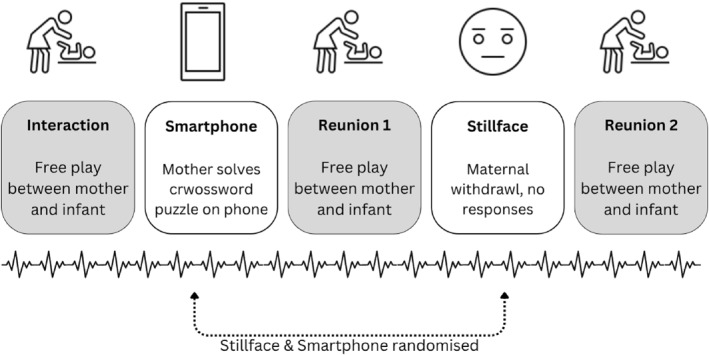
Schematic description of the experimental phases. Behavioral and cardiac data was collected for all phases. Each phase lasted 2 min, unless shortened due to infant distress (e.g., protesting or crying). The still‐face phase and the smartphone phase were presented randomly to avoid sequence effects.

Data for this study were collected between 2019 and 2021 after the study received approval from the ethics committee of the state of Salzburg (415‐E/2357/2‐2018). During the lab visit, mothers first provided background information and reported their smartphone use via self‐report, with mothers retrospectively estimating their average daily use in hours and minutes. Both mothers and children were fitted with portable Bluetooth electrocardiogram (ECG) recorders to monitor their heart rate. Mothers and infants were then observed in a 360‐degree video lab during a modified still‐face paradigm. Infants were seated in an age‐appropriate seat that was secured on a small table while mothers sat in a chair facing them at a distance of approximately 40 cm. The procedure consisted of five phases (see Figure 1): (1) a free‐play interaction condition where mothers were instructed to play with their child without toys. (2) A still‐face condition where mothers were instructed to look above their child without any facial expression or reaction. (3) A smartphone condition where mothers completed a crossword puzzle on a smartphone provided by the experimenter. Mothers were instructed to use the phone as they usually would and were permitted to maintain physical, verbal and visual contact with their child. (4 and 5) Reunion conditions following each the still‐face and smartphone interruption where mothers were instructed to interact with their child again. The order of the still‐face and smartphone conditions was randomized to control for sequence effects and avoid confounding by prior interruptions. Each condition lasted 2 min, except when children were displaying much distress (e.g., protesting or crying), in which case the duration was shortened.

### 2.3 Measures

#### 2.3.1 Cardiac Activity

Cardiac activity was recorded using a lightweight electrocardiac recording device attached to infants' and mothers' chest using two electrodes (FAROS180°, Bittium Biosignals Ltd., Kuopio, Finland). The recording and the analysis of the ECG data was performed by the Visu‐HRV software (Center for Neuroscience Research, Trier, Germany). A modified Pan‐Tompkins algorithm was used to detect the R‐waves from the ECG chest wall lead. The RR intervals (i.e., inter‐beat intervals—IBIs) were segmented into consecutive intervals with a total duration equal to the duration of the observation session. The millisecond‐based IBIs were automatically checked for artifacts and corrected according to the “NEUROCOR precisionHRV Algorithm” guidelines (Wittling 2014). The algorithm marked all IBIs outside of age‐dependent physiological limits. All intervals where the moving average over five consecutive IBIs exceeded an age‐dependent threshold were marked, and replaced by spline‐interpolated IBIs of equivalent length without altering the total duration of the signal. In cases of extended artifact phases, the distribution of R‐wave timings was adjusted to match the R‐wave dynamics before and after the artifact. The percentage value of the artifact load, based on the total number of IBIs and the number of corrected IBIs, was calculated. Additionally, raw and artifact‐corrected IBI data were visually inspected to identify segments with persistent signal irregularities that could not be adequately corrected. Experimental phases with an artifact load > 5% or flagged by visual inspection were excluded (*n* = 34 for mothers; *n* = 56 for children). Shorter IBIs, corresponding to higher heart rates, were interpreted as increased cardiac arousal (and vice versa). RSA was calculated from IBIs using the method of Abney, daSilva, Lewis, and Bertenthal (2021), with higher RSA values reflecting greater parasympathetic activity and physiological regulation.

#### 2.3.2 Behavioral Measures

Infant and maternal behavior were micro‐coded in a frame‐by‐frame interval using Mangold INTERACT (Mangold International GmbH 2017). Two research assistants independently rated 20% of all available videos (*n* = 13) to obtain inter‐rater reliability.

##### 2.3.2.1 Infant Behavior

*Protest* was coded when at least two indicators of protest were present at the same time: (1) grimacing, (2) crying, screaming or whining, and (3) trying to get out of the infant seat (Reck et al. 2009). *Self‐soothing behavior* was operationalized as infant‐initiated oral self‐soothing (i.e., infants suckled on their own body, their caregiver's body, or other items like clothing) and/or self‐touch (i.e., infant hands touching one another; Reck et al. 2009). Inter‐rater reliability was *κ* = 0.84 for protest, and *κ* = 0.89 for self‐soothing behavior.

##### 2.3.2.2 Maternal Behavior

*Soothing touch* was defined as affectionate kissing, stroking or massaging (Jean and Stack 2009). Statically placing the palm on the infant or holding the infant were also coded, unless accompanied by stimulating touch. Rough, intrusive, stimulating and functional touches were excluded. *Gaze* was coded as maternal social gaze towards their infants' faces. *Vocalizations* were operationalized as positive or neutral. Positive vocalizations included infant‐directed speech and other communicative sounds. Inter‐rater reliability was *κ* = 0.70 for soothing touch, *κ* = 0.81 for maternal gaze, and *κ* = 0.90 for vocalizations.

### 2.4 Statistical Analyses

We analyzed the effects of smartphones on maternal and infants' physiological and behavioral dynamics using linear mixed‐effects modeling (LMM; lme4 package in R; Bates et al. 2015) and dynamic structural equation modeling (DSEM; Mplus 8.8; Muthén and Muthén 2018). Outliers, defined as values exceeding 1.5 times the interquartile distance below the first or above the third quartile, were retained after verifying their plausibility.

#### 2.4.1 Linear Mixed‐Effects Modeling

To test phase‐based changes in maternal and infant behavior and cardiac activity, we fitted separate LMMs for each dependent variable (i.e., maternal and infant behavior, cardiac activity) with phase (baseline, smartphone interruption, reunion after smartphone, still‐face interruption, or reunion after still‐face) as a fixed effect and participant ID as a random intercept. Estimated marginal means (EMM) and pairwise comparisons were computed to interpret significant effects. Further, we examined whether mothers' everyday smartphone use predicted social engagement during the smartphone reunion phase, by computing separate LMMs with the various maternal behaviors as outcome and daily smartphone use as a predictor, including participant ID as a random intercept. In exploratory LMMs we tested whether maternal habitual smartphone use predicted infants' protest, self‐regulation, and cardiac arousal during the smartphone phase. For the LMMs, missing data were handled through listwise deletion, such that any observation with missing values on the outcome or predictors was excluded from the analysis.

#### 2.4.2 Dynamic Structural Equation Modeling

To explore temporal coupling effects in mother–infant dyads, we employed DSEM to model dynamic processes over time. We estimated time‐lagged associations at 1‐s resolution between maternal and infant behaviors and cardiac activity, allowing us to examine whether changes in maternal behaviors at time *t* − 1 predicted changes in infant behavior or arousal at time *t*, and vice versa. Due to model complexity, separate models were computed for each interaction phase. In analyses focusing on the links between maternal and infant behaviors, models were further run separately for each maternal behavior (soothing touch, social gaze, and positive vocalizations).

Models were estimated using Bayesian estimation with uninformative priors and a default maximum of 50,000 Markov Chain Monte Carlo (MCMC) iterations. Missing data were addressed using a Kalman filter, allowing all available data to contribute to parameter estimation. In DSEM, the Kalman filter uses predicted values as an estimate of missing values within a time‐series. This method preserves all observations, even when many time points have missing outcomes or predictors (see McNeish and Hamaker 2020). Simulation studies further demonstrate that DSEM performs reliably with up to 90% missing data (Asparouhov et al. 2018). Convergence was assessed using Potential Scale Reduction (PSR) values, with thresholds below 1.1 indicating acceptable convergence. Once convergence was reached, iterations were doubled to confirm stability. To facilitate model estimation, all variables were treated as continuous, as specifying them as binary prevented DSEM from converging. This approach allowed stable estimation of dynamic effects while preserving the temporal structure of the data. Parameter significance was evaluated via 95% credible intervals; effects whose intervals excluded zero were interpreted as statistically meaningful.

## 3 Results

Descriptive statistics for all outcome variables in all phases of the experiment are displayed in Table 2.

**TABLE 2:** Descriptive statistics for infant and maternal behavior (percentage) and cardiac arousal (inter‐beat‐intervals in ms).

	Baseline interaction	Smartphone	Reunion after smartphone	Still‐face	Reunion after still‐face
Infant protest					
Mean (SD)	7.43 (14.36)	27.97 (26.22)	20.32 (23.22)	32.39 (25.89)	22.67 (28.01)
Min–max	0–66.50	0–93.11	0–83.13	0–92.21	0–100
Infant self‐soothing					
Mean (SD)	22.24 (24.03)	17.56 (23.27)	24.66 (25.11)	22.03 (20.34)	18.82 (21.67)
Min–max	0–100	0–94.89	0–94.60	0–84.23	0–80.79
Infant cardiac arousal					
Mean (SD)	438.03 (46.71)	422.40 (37.94)	427.94 (43.90)	420.75 (43.60)	422.27 (45.48)
Min–max	204.00–645.50	302.86–585.28	288.89–645.50	320.47–667.75	301.71–669.19
Infant RSA					
Mean (SD)	3.66 (1.02)	3.39 (0.91)	3.55 (1.03)	3.57 (1.02)	3.55 (0.98)
Min–max	0.73–7.38	0.62–6.60	0.80–6.87	1.03–7.47	0.85–6.60
Maternal soothing touch					
Mean (SD)	17.92 (18.61)	14.90 (21.64)	19.24 (19.06)	0	21.09 (18.64)
Min–max	0–76.77	0–86.96	0–90.36	0	0–92.01
Maternal social gaze					
Mean (SD)	88.30 (9.22)	8.39 (8.43)	87.53 (8.19)	0	87.83 (10.42)
Min–max	59.32–100	0–37.42	57.88–97.32	0	50.71–99.38
Maternal vocalization					
Mean (SD)	88.49 (11.80)	31.63 (26.74)	88.94 (11.72)	0	89.09 (9.69)
Min–max	51.63–100	0–100	40.35–100	0	57.02–99.03
Maternal cardiac arousal					
Mean (SD)	709.17 (118.77)	743.37 (122.45)	726.85 (102.46)	753.78 (116.21)	732.10 (110.28)
Min–max	212.00–1222.54	473.85–1402.40	441.59–1166.72	424.00–1106.82	480.00–1310.82
Maternal RSA					
Mean (SD)	5.61 (1.04)	5.72 (1.04)	5.58 (1.04)	5.67 (1.05)	5.48 (1.12)
Min–max	2.11–8.77	1.32–10.20	1.86–10.4	2.27–8.33	1.85–9.35

### 3.1 Infant Experience of Interruptions During Social Interactions

**FIGURE 2:**
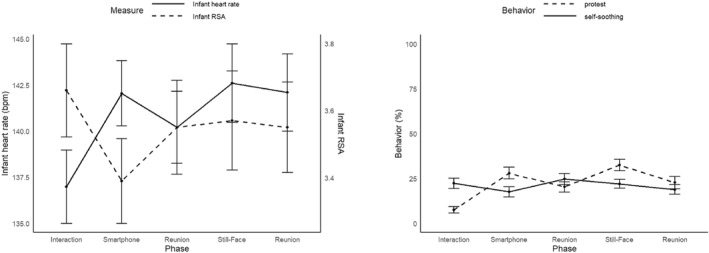
Infant autonomic activity and behavior across experimental phases. Error bars show standard error. The still‐face phase and the smartphone phase were presented randomly to avoid sequence effects.

In line with our hypotheses, infants exhibited significantly more protest behavior during the smartphone interruption (EMM = 0.26) compared to the interaction baseline (EMM = 0.06, *p* < 0.001). In addition, infants showed significantly higher cardiac arousal (i.e., lower IBIs) during the smartphone phase (EMM = 425) than during baseline (EMM = 438, *p* < 0.001), indicating increased physiological stress during the smartphone interruption. Infants' RSA was significantly lower during the smartphone interruption (EMM = 3.54) compared to the interaction baseline (EMM = 3.63, *p* < 0.001), suggesting a withdrawal of parasympathetic regulation in the context of parental unavailability. Contrary to our expectations, infants displayed significantly less self‐regulatory behavior during the smartphone interruption (EMM = 0.1) compared to baseline (EMM = 0.22, *p* < 0.001). Furthermore, infants displayed significantly more protest (EMM = 0.32, *p* < 0.001) and self‐regulatory behavior (EMM = 0.21, *p* < 0.001) as well as higher cardiac arousal (EMM = 420, *p* < 0.001) during the still‐face compared to the smartphone phase. In contrast, RSA was significantly lower during the smartphone phase (EMM = 3.46) compared to the still‐face phase (EMM = 3.54, *p* < 0.001). See Figure 2 and Supporting Information S1: Table S1 for detailed results of the mixed effects models.

Maternal habitual smartphone use showed no significant association with protest behavior (*b* = −0.028, SE = 0.029, *t*(58.50) = −0.96, *p* = .340), nor with self‐regulatory behaviors (*b* = 0.013, SE = 0.026, *t*(59.02) = 0.51, *p* = .613) during the smartphone phase.

### 3.2 Maternal Engagement During Interruptions of Social Interactions

There was a significant main effect of phase on maternal social gaze (*p* < 0.001). Specifically, mothers used less social gaze during the smartphone (EMM = 0.07) and still‐face interruptions (EMM = 0.02) compared to the interaction baseline (EMM = 0.88, *p* < 0.001). No significant differences were observed between baseline and both the smartphone (EMM = 0.87, *p* = 0.28) and the still‐face reunion (EMM = 0.88, *p* = 0.86). Additionally, social gaze levels were similar during both reunion phases (*p* = 0.89). These findings support the hypothesis that maternal gaze decreases substantially during smartphone use but returns to baseline during reunions.

Results also revealed a significant main effect of phase on maternal touch (*p* < 0.001). Touch decreased significantly from baseline (EMM = 0.18) to both the still‐face interruption (EMM = 0.01, *p* < 0.001), and, to a lesser extent, the smartphone interruption (EMM = 0.15, *p* < 0.001). During the reunion phase after the still‐face interruption, touch increased significantly relative to baseline (EMM = 0.21; *p* < 0.001). No such effect was observed during the reunion phase following the smartphone interruption where maternal touch returned to baseline levels (EMM = 0.19; *p* = 0.37). Touch during the still‐face reunion was significantly higher than during the smartphone reunion (*p* = 0.001). This pattern indicates a recovery and slight enhancement of tactile engagement after interruptions, especially following the still‐face phase.

Maternal vocalizations varied significantly across phases (*p* = 0.01). Vocalizations were substantially reduced during both smartphone (EMM = 0.31) and still‐face (EMM = 0.02) interruptions compared to baseline (EMM = 0.88, both *p*s < 0.001). Vocalizations during both the smartphone reunion (EMM = 0.89) as well as the still‐face reunion (EMM = 0.89) did not differ significantly from baseline (*p* = 0.08), but vocalizations were significantly more frequent during still‐face reunion compared to smartphone reunion (*p* = 0.01). These results demonstrate a strong suppression of vocal behavior during interruptions and recovery during reunions, particularly following the still‐face phase.

**FIGURE 3:**
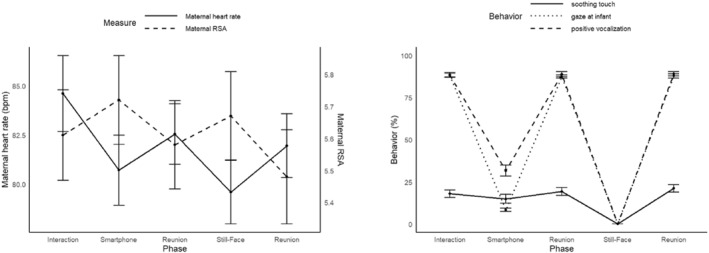
Maternal autonomic activity and behavior across experimental phases. Error bars show standard error. The still‐face phase and the smartphone phase were presented randomly to avoid sequence effects.

Maternal cardiac arousal and RSA varied significantly with phase (both *p*s < 0.001). Cardiac arousal was lowest during the still‐face interruption (EMM = 760) and reduced during smartphone interruption (EMM = 747) compared to the baseline interaction phase (EMM = 704, both *p*s < 0.001). Cardiac arousal was significantly higher during the reunions after both the smartphone (EMM = 727) and the still‐face (EMM = 732) compared to the actual interruption phases, respectively (both *p*s < 0.01), but remained reduced relative to the interaction baseline. RSA was higher during both the smartphone (EMM = 5.72) and still‐face interruptions (EMM = 5.71) compared to the interaction baseline (EMM = 5.62, both *ps* < 0.001), In contrast, RSA decreased during the smartphone (EMM = 5.57) and still‐face reunions (EMM = 5.52) relative to the interaction baseline (both *ps* < 0.001), showing a partial recovery but remaining lower than during the preceding interruptions (both *ps* < 0.001). See Figure 3 and Supporting Information S1: Table S2 for detailed results of the mixed effects models.

Everyday smartphone use was not significantly related to maternal touch (*b* = −0.014, SE = 0.023, *t*(60.08) = −0.61, *p* = 0.543), social gaze (*b* = −0.011, SE = 0.009, *t*(58.58) = −1.17, *p* = 0.246), nor positive vocalizations (*b* = −0.053, SE = 0.028, *t*(59.45) = −1.90, *p* = 0.063), suggesting that overall maternal engagement was largely unaffected by habitual smartphone use.

### 3.3 Dyadic Effects Between Mothers and Infants

Examining the dynamic effects between mothers' and infants' behaviors, results showed that during the baseline interaction phase, increased maternal vocalizations at time *t* − 1 were associated with reduced infant protest at time *t* (*b* = −0.037, CI = −0.057 to −0.017). There were no significant effects of maternal gaze and soothing touch on infants' behaviors. Similarly, there were no significant effects of maternal touch, vocalizations and social gaze on child behaviors during the smartphone interruption. Full results of these models are reported in Supporting Information S1: Table S3.

For models involving measures of cardiac arousal and parasympathetic activity, we encountered substantial estimation difficulties. For cardiac arousal, reliable estimation was achieved only when restricting models to the estimation of fixed effects at the population level, thus capturing average coupling patterns while omitting individual‐level variation. We found no significant effects of maternal social behaviors on infant cardiac arousal during any of the phases (see Supporting Information S1: Table S4). In contrast, there was evidence for HR coupling such that during the still‐face phase, low maternal cardiac arousal at time *t* − 1 was associated with increased infant cardiac arousal at time *t* (*b* = −0.021, CI = −0.037 to −0.005), and low infant cardiac arousal at *t* − 1 was associated with increased maternal cardiac arousal at *t* (*b* = −0.033, CI = −0.047 to −0.019). During the smartphone phase, decrease in infant cardiac arousal at *t* − 1 was significantly associated with increased maternal cardiac arousal at *t* (*b* = −0.029, CI = −0.045 to −0.013), but not vice versa. No significant effects were observed during the interaction nor the reunion phases. For full results, see Supporting Information S1: Table S5. RSA estimation proved more challenging; despite testing multiple modeling strategies, convergence was not reliably achieved and parameter estimates were unstable, rendering them uninterpretable. Therefore, RSA model results are not reported.

### 3.4 Power Analysis

Post hoc power analyses were conducted using GPower 3.1.9.7 for models examining infant and maternal behaviors and physiological responses across the experimental phases. For each model, the total sample size, number of predictors, and observed *R* ^2^ were used to estimate statistical power (*α* = 0.05). For these models we obtained power values between 0.80 and 1, suggesting sufficient power to detect effects of the observed magnitude. For models additionally including habitual smartphone use as a predictor, we obtained power values between 0.434 and 0.999, indicating that all but one model (i.e., maternal social gaze) had sufficient power to detect effects of the observed magnitude. As G*Power does not support mixed‐effects modeling, power estimates are based on fixed‐effects approximations and may slightly overestimate true model power due to the omission of random effect variance.

## 4 Discussion

This study employed a multi‐method approach to investigate how maternal smartphone use may disrupt early social interactions, affecting both infant and maternal behavior as well as physiology. During the smartphone interruption, infants displayed increased protest and heightened cardiac arousal but reduced parasympathetic activity and self‐regulatory behaviors compared to the baseline interaction. Mothers showed significantly reduced social gaze, touch, and vocalizations during both interruption phases, and they re‐engaged in these interactive behaviors in a more pronounced way following the still‐face than after smartphone use. Maternal HR decreased during both interruption phases and only partially recovered during the respective reunions, while RSA increased during both interruptions and decreased during both reunion phases. Time‐lagged analyses revealed dynamic behavioral associations between maternal vocalizations and infant protest during the interaction phase. We also found physiological coupling between maternal and infant cardiac activity during both interruption conditions, suggesting a dynamic interplay between their physiological systems. These findings provide experimental evidence on the immediate effects of smartphone use during mother–infant interactions. By integrating behavioral and physiological measures, this study addresses recent calls for examining short‐term stress responses beyond cognitive and socioemotional outcomes (Toledo‐Vargas et al. 2025), and further illuminates how technoference disrupts moment‐to‐moment co‐regulation within caregiver‐infant dyads.

In line with our hypotheses, infants displayed significantly more protest behavior and heightened cardiac arousal, alongside reduced parasympathetic activity, during the smartphone interruption compared to baseline, consistent with prior research suggesting that technoference can serve as a relational stressor (e.g., Mikić and Klein 2022; Rozenblatt‐Perkal et al. 2022; Stockdale et al. 2020). Similar to classic still‐face studies, where infants show distress and vagal suppression in response to parental unavailability (Mesman et al. 2009; Moore and Calkins 2004), these results support the view that parental smartphone use may function as modern still‐face (e.g., Braune‐Krickau et al. 2021; Mikić and Klein 2022), eliciting infant dysregulation while caregiver co‐regulation is disrupted. Surprisingly, infants in this study showed less self‐regulatory behavior during the smartphone interruption compared to baseline, contrasting classical still‐face studies (Mesman et al. 2009) and recent findings on smartphone interruptions reporting increased self‐comforting (e.g., Stockdale et al. 2020). In contrast to previous studies, where caregivers were prohibited from interacting with their infants, mothers in our study were allowed to vocalize and touch their infants, potentially reducing infant distress (Feldman et al. 2010). Still, the still‐face elicited more protest and self‐regulatory behavior than the smartphone interruption, suggesting that both were upsetting but in different ways (Stallworthy et al. 2024). This aligns with evidence that infants respond differently when interactional disruptions have a visible cause (Legerstee and Markova 2007), suggesting that maternal smartphone use may have been perceived as less socially ambiguous than the still‐face. Although upsetting, the visible engagement with the phone may have signaled a temporary break, reducing the need for active self‐regulation attempts, whereas the unexplained, complete withdrawal during the still‐face likely posed a stronger stressor, prompting both infant protest and self‐soothing efforts (Mesman et al. 2009). Including a control condition with a less disruptive or ambiguous interruption (e.g., reading a book) could have enabled a more precise comparison. Yet, contrasting the smartphone interruption with the still‐face provides a conservative test, given the well‐documented robustness of infants' responses to the still‐face.

As expected, maternal interactive behavior (i.e., social gaze, touch, vocalizations) decreased substantially during the smartphone interruption, consistent with previous work showing that technoference reduces parental attention and engagement attempts (see Braune‐Krickau et al. 2021; Mikić and Klein 2022, for reviews). In reunion phases after both interruptions gaze levels recovered, indicating that gaze is a key channel mothers use to re‐engage following relational disruptions (Kim et al. 2014; MacLean et al. 2014). Interestingly, maternal tactile and vocal behaviors showed a different pattern. While touch and vocalizations also recovered in the reunions, both were significantly higher during the still‐face reunion than during the smartphone reunion. This suggests that although mothers re‐engage after both types of interruptions (Konrad, Hillmann, et al. 2021), their efforts seem to be more pronounced following the still‐face, possibly reflecting its more artificial and uncomfortable nature. Consistently, the still‐face also elicited greater infant protest and self‐regulatory behavior than the smartphone‐interruption, indicating that the still‐face represents a stronger relational stressor. Thus, mothers may have been more attuned to the relational rupture caused by the still‐face and responded with more active repair efforts, whereas the smartphone interruption may have been experienced as less disruptive, prompting less compensatory re‐engagement (see also Braune‐Krickau et al. 2021). Maternal parasympathetic activity increased while cardiac arousal decreased during both interruptions, potentially reflecting a more regulated state, withdrawal, reduced affective engagement, or a shift in attentional focus (Oppenheimer et al. 2013). Maternal cardiac arousal only partially recovered during both reunions and remained lower than during interaction baseline. Meanwhile, maternal parasympathetic activity decreased during both reunions, suggesting context‐sensitive adjustment possibly indicating a calmer, more focused state during both interruptions and re‐engagement with the infant after disruptions. These patterns align with previous studies showing increased maternal RSA during brief social withdrawal to maintain attentional and affective resources, and decreased RSA during reunion as part of active regulatory engagement (Moore and Calkins 2004; Moore et al. 2009; Oppenheimer et al. 2013; Conradt and Ablow 2010). Given that heart rate is a nonspecific physiological marker, these findings should be interpreted with caution, and no firm conclusions can be drawn regarding a potential relation to maternal social engagement.

Finally, we found no associations between maternal habitual smartphone use and infant protest nor self‐regulation, contrasting evidence showing reduced positive affect and re‐engagement with higher parental smartphone use (Konrad, Hillmann, et al. 2021; Myruski et al. 2018; Stockdale et al. 2020). Still, these null results align with other work reporting no relationship between caregiver‐reported technoference and infants' negative affect during smartphone interruptions (e.g., Rozenblatt‐Perkal et al. 2022). Similarly, we found no associations between smartphone use and maternal behavior during or after the smartphone interruption further suggesting that habitual use levels alone may not reliably predict dyadic responses during structured, standardized interactions. Notably, parental attitudes toward technoference (i.e., how acceptable they judged phone use in interactions) were stronger predictors of infant behavior than self‐reported frequency of technoference itself (Stockdale et al. 2020). Therefore, it is possible that mothers who hold particularly negative attitudes toward smartphone use during interactions with their infants are more likely to participate in studies examining this dynamic. Another possibility is that, because mothers in our paradigm were allowed to continue interacting with their infants during the smartphone phase, those with higher levels of habitual use may have been more practiced at balancing both a phone‐based task and child engagement, reducing the likelihood of detectable differences in our setting. Moreover, recent work points out that self‐reported overall use may poorly capture situational phone use patterns specifically during child‐focused interactions (Molaib et al. 2025). Prior studies have used diverse measures of habitual smartphone use. Some employed standardized tools like the CAFE Media Assessment Questionnaire (e.g., Konrad, Berger‐Hanke, et al. 2021), while others relied on self‐developed questionnaires assessing daily use and duration (Myruski et al. 2018), or reported technoference during parent–child interactions (Stockdale et al. 2020; Rozenblatt‐Perkal et al. 2022). This heterogeneity in measurement approaches likely contributes to inconsistencies in findings and complicates comparisons across studies. Taken together, our findings suggest that habitual use patterns may be less predictive of maternal behavior in structured settings than moment‐to‐moment dynamics, pointing to the need for a closer examination of real‐time parent‐infant coordination during smartphone use.

Time‐lagged analyses revealed that increased maternal vocalizations predicted less infant protest at a 1‐s lag during the baseline interaction phase. This finding corroborates previous research suggesting that vocalizations play an important role in modulating infant affective states (e.g., Provenzi et al. 2018), as they provide infants with important cues about maternal availability. Interestingly, we also found bidirectional coupling of maternal and infant cardiac arousal during the still‐face interruption, indicating that lower maternal arousal predicted increased infant arousal at a 1‐s lag and vice versa. While some studies report physiological synchrony or coupling under conditions of interactional stress (e.g., Busuito et al. 2019), this inverse pattern may reflect a breakdown in co‐regulation under relational stress (see Abney, daSilva, and Bertenthal 2021b). That is, when maternal arousal drops—potentially signaling emotional withdrawal or disengagement—the infant's arousal rises in the absence of co‐regulatory support, reflecting heightened stress or protest. Conversely, when the infant’s arousal decreases, the mother's arousal increases, which might indicate a lagged or asynchronous physiological adjustment rather than coordinated regulation. In contrast to the still‐face phase, during the smartphone interruption, only reductions in infant arousal were associated with increases in maternal arousal, while no reciprocal effects were found. This asymmetry suggests a disruption in mutual physiological attunement, consistent with prior work indicating that parental technoference reduces parents' capacity for real‐time, reciprocal regulation (McDaniel and Radesky 2018). Notably, Stallworthy et al. (2024), using a bivariate vector autoregressive model, reported positive RSA synchrony between mothers and infants during a technoference interruption, suggesting that physiological coupling may vary depending on the specific autonomic marker assessed (e.g., RSA vs. IBI), interactional context, or dyadic characteristics. Due to persistent model convergence difficulties preventing reliable estimation of RSA coupling, we were unable to replicate Stallworthy et al. ’s (2024) results. This underscores the need to clarify the physiological and contextual factors that shape reciprocal co‐regulation during everyday interactions and to address methodological challenges associated with complex computational approaches such as DSEM. Model convergence and stability appear highly sensitive to data structure, model specification, and examined variables, highlighting the importance of understanding the conditions for robust and interpretable estimation.

This study has several strengths including its multi‐method approach, combining behavioral observations with physiological measures of cardiac activity, as well as frame‐by‐frame behavioral coding paired with continuous ECG recording enabling detailed time‐lagged analyses of dynamic mother–infant interaction processes. However, certain limitations must be acknowledged. First, while the laboratory setting ensured experimental control, it inevitably reduced ecological validity. While this was necessary to be able to isolate the effects of smartphone presence from confounding variables and ensure consistency across participants, the use of a standardized, muted smartphone without notifications or personal content likely made the interruption less engaging than a mother's own device would be in everyday contexts. Although we attempted to control for maternal habitual smartphone use, it was assessed via a self‐report measure, which potentially differs from actual screen times (Molaib et al. 2025) and offers only a rough estimate of usage patterns that may be subject to recall bias or social desirability effects. Another limitation concerns the short‐term nature of the observed effects. This study focused on immediate behavioral and physiological responses to interruptions, without assessing potential longer‐term consequences for infant stress regulation, socio‐emotional development, or relational outcomes. Previous research found associations between parental media use and children’s later executive functioning or language development (Gaudreau et al. 2022; A. J. Morris et al. 2022; Yang et al. 2023), but future longitudinal studies are needed to investigate whether repeated experiences of technoference during early interactions contribute to lasting effects on infant socio‐emotional development. It would be particularly important to consider the role of elevated cardiac arousal and reduced maternal re‐engagement behaviors as potential mechanisms underlying these developmental trajectories. A key limitation of this study is the inability to reliably estimate RSA coupling due to persistent convergence difficulties. These likely stem from limited between‐person variation, complex missing data patterns, and non‐stationarity of data, which also partially affected DSEM models for cardiac arousal and behavior. For example, random effects could not be estimated for HR models, and behavioral variables had to be treated as continuous to ensure convergence. Such compromises may limit the validity of parameter estimates and highlight broader methodological challenges associated of complex models like DSEM. Future work needs to clarify the conditions under which these models yield stable, reliable, and interpretable estimates. Furthermore, the study's exclusive focus on mothers limits the generalizability of the findings, as it remains unclear whether similar patterns would emerge in fathers or other caregivers. Given evidence for gender differences in caregiving behavior (Yaffe 2023) and stress regulation (Adjei et al. 2018), this represents an important direction for future research. Lastly, the homogeneity of the sample, with relatively high maternal education levels, may restrict the generalizability of the findings to more diverse populations. Future studies should therefore aim to include more socioeconomically and culturally diverse samples to better capture the variability present in real‐world parenting contexts.

A growing body of evidence on the disruptive effects of parental smartphone use on early caregiver‐child interactions highlights an urgent need to identify practical strategies to support family interaction quality in the digital age. Consistent with recent meta‐analytic findings showing associations between parental technology use and poorer child outcomes, including lower attachment security, diminished cognitive skills, and increased psychosocial difficulties (Toledo‐Vargas et al. 2025), our results emphasize the importance of promoting responsive and engaged caregiving, particularly during early infancy when stress regulation capacities are still developing. In line with Braune‐Krickau et al. (2021), future research must move beyond documenting risk and begin exploring protective mechanisms and intervention strategies aimed at minimizing the consequences of technoference and parental absorption through smartphones. This could include promoting mindful and intentional smartphone use practices among parents, developing interventions that address both behavioral patterns and the emotional needs underlying smartphone reliance, and fostering public awareness about the potential relational and developmental risks of frequent interruptions. Additionally, longitudinal and ecologically valid studies in everyday family contexts are needed to assess whether and how repeated smartphone interruptions may shape early stress regulation, children's cognitive and emotional development and relational processes over time. Ultimately, future research should aim to clarify in which situations and under which relational conditions parental smartphone use becomes developmentally consequential, and how these risks might be mitigated through media education, parental awareness, and targeted, family‐centered interventions.

## Author Contributions

**Antonia Dinzinger:** conceptualization, methodology, investigation, funding acquisition, project administration, writing – original draft, writing – review and editing. **Elke Greif:** data curation, formal analysis, visualization, writing – original draft. **Lydia Speyer:** software, formal analysis, visualization, writing – original draft. **Ralf Arne Wittling:** software, writing – review and editing. **Karl Heinz Brisch:** methodology, funding acquisition, project administration, writing – review and editing, **Beate Priewasser:** supervision, resources, writing – review and editing. **Gabriela Markova:** conceptualization, methodology, supervision, writing – original draft, writing – review and editing.

## Ethics Statement

The study received approval from the ethics committee of the Land Salzburg (415‐E/2357/2‐2018).

## Conflicts of Interest

The authors declare no conflicts of interest.

## Supporting information


Supporting Information S1

## Data Availability

The dataset is available from the corresponding author upon reasonable request.
