# Supplementary material for: Tiny Screens, Big Impact: Effects of Maternal Smartphone Use on Maternal and Infants' Physiological and Behavioral Stress and Interaction Dynamics
Source: Infancy. 2025 Dec 6;30(6):e70056. doi: 10.1111/infa.70056 (PMC12681296; doi:10.1111/infa.70056)
Supplement: Supplementary file 1 — Supporting Information S1 [file INFA-30-0-s001.docx]

**Supplementary Materials**

Table S1.

*Results of the linear mixed effects models examining differences in infant behavior and cardiac arousal across experimental phases.*

| **Comparison** | ***β*** | ***SE*** | ***Z*** | ***p*** | ***EMM_1_*** | ***EMM_2_*** |
| --- | --- | --- | --- | --- | --- | --- |
| Protest Behaviour: Smartphone_1_ vs. Interaction_2_ | -0.20 | 0.01 | 36.92 | < .001 | 0.26 | 0.06 |
| Self-regulatory Behavior: Smartphone_1_ vs. Interaction_2_ | 0.04 | 0.01 | -7.02 | < .001 | 0.18 | 0.22 |
| Cardiac Arousal: Smartphone_1_ vs. Interaction_2_ | 13.82 | 0.64 | -21.79 | < .001 | 425 | 438 |
| RSA: Smartphone_1_ vs. Interaction_2_ | 0.08 | 0.01 | 6.18 | < .001 | 3.54 | 3.63 |
| Protest Behaviour: Smartphone_1_ vs. Still-face _2_ | -0.05 | 0.01 | -6.57 | < .001 | 0.28 | 0.32 |
| Self-regulatory Behavior: Smartphone_1_ vs. Still-face_2_ | -0.04 | 0.01 | -6.74 | < .001 | 0.17 | 0.21 |
| Cardiac Arousal: Smartphone_1_ vs. Still-face _2_ | 2.03 | 0.64 | -3.19 | .001 | 422 | 420 |
| RSA: Smartphone_1_ vs. Still-face _2_ | -0.09 | 0.02 | -5.81 | < .001 | 3.46 | 3.54 |

Table S2.

*Results of the linear mixed effects models examining differences in maternal behavior and cardiac arousal across experimental phases.*

| **Comparison** | ***β*** | ***SE*** | ***Z*** | ***p*** | ***EMM_1_*** | ***EMM_2_*** |
| --- | --- | --- | --- | --- | --- | --- |
| Outcome: Maternal Social Gaze |  |  |  |  |  |  |
| Interaction_1_ vs. Smartphone Reunion_2_ | 0.01 | 0.05 | 1.95 | 0.29 | 0.88 | 0.87 |
| Interaction_1_ vs. Still-face Reunion_2_ | 0.05 | 0.05 | 0.99 | 0.86 | 0.88 | 0.88 |
| Interaction_1_ vs. Smartphone_2_ | 0.80 | 0.05 | 168.99 | <.001 | 0.88 | 0.07 |
| Interaction_1_ vs. Still-face_2_ | 0.86 | 0.05 | 180.74 | <.001 | 0.88 | 0.02 |
| Smartphone Reunion_1_ vs. Still-face Reunion_2_ | -0.01 | 0.05 | -0.91 | 0.89 | 0.87 | 0.88 |
| Smartphone Reunion_1_ vs. Smartphone_2_ | 0.80 | 0.05 | 165.21 | <.001 | 0.87 | 0.07 |
| Smartphone Reunion_1_ vs. Still-face_2_ | 0.85 | 0.05 | 175.87 | <.001 | 0.87 | 0.02 |
| Still-face Reunion_1_ vs. Smartphone_2_ | 0.80 | 0.05 | 162.02 | <.001 | 0.88 | 0.07 |
| Still-face Reunion_1_ vs. Still-face_2_ | 0.86 | 0.05 | 174.78 | <.001 | 0.88 | 0.02 |
| Smartphone_1_ vs. Still-face_2_ | 0.06 | 0.05 | 11.36 | <.001 | 0.07 | 0.02 |
|  |  |  |  |  |  |  |
| Outcome: Maternal Soothing Touch |  |  |  |  |  |  |
| Interaction_1_ vs. Smartphone Reunion_2_ | -0.01 | 0.01 | -1.80 | .37 | 0.18 | 0.19 |
| Interaction_1_ vs. Still-face Reunion_2_ | -0.03 | 0.01 | -5.65 | <.001 | 0.18 | 0.21 |
| Interaction_1_ vs. Smartphone_2_ | 0.03 | 0.01 | 5.68 | <.001 | 0.18 | 0.15 |
| Interaction_1_ vs. Still-face_2_ | 0.17 | 0.01 | 30.88 | <.001 | 0.18 | 0.01 |
| Smartphone Reunion_1_ vs. Still-face Reunion_2_ | -0.02 | 0.01 | -3.83 | .001 | 0.19 | 0.21 |
| Smartphone Reunion_1_ vs. Smartphone_2_ | 0.04 | 0.01 | 7.36 | <.001 | 0.19 | 0.15 |
| Smartphone Reunion_1_ vs. Still-face_2_ | 0.18 | 0.01 | 32.10 | <.001 | 0.19 | 0.01 |
| Still-face Reunion_1_ vs. Smartphone_2_ | 0.06 | 0.01 | 10.92 | <.001 | 0.21 | 0.15 |
| Still-face Reunion_1_ vs. Still-face_2_ | 0.20 | 0.01 | 35.54 | <.001 | 0.21 | 0.01 |
| Smartphone_1_ vs. Still-face_2_ | 0.14 | 0.01 | 24.30 | <.001 | 0.15 | 0.01 |
|  |  |  |  |  |  |  |
| Outcome: Maternal Positive Vocalizations |  |  |  |  |  |  |
| Interaction_1_ vs. Smartphone Reunion_2_ | -0.004 | 0.01 | -0.84 | .92 | 0.88 | 0.89 |
| Interaction_1_ vs. Still-face Reunion_2_ | -0.02 | 0.01 | -3.29 | .01 | 0.88 | 0.90 |
| Interaction_1_ vs. Smartphone_2_ | 0.57 | 0.01 | 110.91 | <.001 | 0.88 | 0.31 |
| Interaction_1_ vs. Still-face_2_ | 0.87 | 0.01 | 168.31 | <.001 | 0.88 | 0.02 |
| Smartphone Reunion_1_ vs. Still-face Reunion_2_ | -0.01 | 0.01 | -2.43 | .11 | 0.89 | 0.90 |
| Smartphone Reunion_1_ vs. Smartphone_2_ | 0.57 | 0.01 | 110.52 | <.001 | 0.89 | 0.31 |
| Smartphone Reunion_1_ vs. Still-face_2_ | 0.87 | 0.01 | 166.30 | <.001 | 0.89 | 0.02 |
| Still-face Reunion_1_ vs. Smartphone_2_ | 0.59 | 0.01 | 110.09 | <.001 | 0.90 | 0.31 |
| Still-face Reunion_1_ vs. Still-face_2_ | 0.88 | 0.01 | 166.90 | <.0001 | 0.90 | 0.02 |
| Smartphone_1_ vs. Still-face_2_ | 0.30 | 0.01 | 55.36 | <.0001 | 0.31 | 0.02 |
|  |  |  |  |  |  |  |
| Outcome: Maternal Cardiac Arousal |  |  |  |  |  |  |
| Interaction_1_ vs. Smartphone Reunion_2_ | -22.88 | 1.23 | -18.65 | <.001 | 704 | 727 |
| Interaction_1_ vs. Still-face Reunion_2_ | -27.07 | 1.26 | -21.55 | <.001 | 704 | 732 |
| Interaction_1_ vs. Smartphone_2_ | -42.44 | 1.24 | -34.10 | <.001 | 704 | 747 |
| Interaction_1_ vs. Still-face_2_ | -55.92 | 1.24 | -45.15 | <.001 | 704 | 760 |
| Smartphone Reunion_1_ vs. Still-face Reunion_2_ | -4.19 | 1.23 | -3.42 | .01 | 727 | 732 |
| Smartphone Reunion_1_ vs. Smartphone_2_ | -19.56 | 1.22 | -16.07 | <.001 | 727 | 747 |
| Smartphone Reunion_1_ vs. Still-face_2_ | -33.04 | 1.21 | -27.34 | <.001 | 727 | 760 |
| Still-face Reunion_1_ vs. Smartphone_2_ | -15.36 | 1.26 | -12.24 | <.001 | 732 | 747 |
| Still-face Reunion_1_ vs. Still-face_2_ | -28.85 | 1.23 | -23.44 | <.001 | 732 | 760 |
| Smartphone_1_ vs. Still-face_2_ | -13.48 | 1.24 | -10.88 | <.001 | 747 | 760 |
|  |  |  |  |  |  |  |
| Outcome: Maternal RSA |  |  |  |  |  |  |
| Interaction_1_ vs. Smartphone Reunion_2_ | 0.05 | 0.01 | 3.95 | <.001 | 5.62 | 5.57 |
| Interaction_1_ vs. Still-face Reunion_2_ | 0.10 | 0.01 | 8.22 | <.001 | 5.62 | 5.52 |
| Interaction_1_ vs. Smartphone_2_ | -0.10 | 0.01 | -8.61 | <.001 | 5.62 | 5.72 |
| Interaction_1_ vs. Still-face_2_ | -0.10 | 0.01 | -8.49 | <.001 | 5.62 | 5.71 |
| Smartphone Reunion_1_ vs. Still-face Reunion_2_ | 0.05 | 0.01 | 4.39 | <.001 | 5.57 | 5.52 |
| Smartphone Reunion_1_ vs. Smartphone_2_ | -0.15 | 0.01 | -12.75 | <.001 | 5.57 | 5.72 |
| Smartphone Reunion_1_ vs. Still-face_2_ | -0.15 | 0.01 | -12.63 | <.001 | 5.57 | 5.71 |
| Still-face Reunion_1_ vs. Smartphone_2_ | -0.20 | 0.01 | -16.81 | <.001 | 5.52 | 5.72 |
| Still-face Reunion_1_ vs. Still-face_2_ | -0.20 | 0.01 | -16.93 | <.001 | 5.52 | 5.71 |
| Smartphone_1_ vs. Still-face_2_ | 0.002 | 0.01 | 0.19 | .99 | 5.72 | 5.71 |

Table S3.

*Parameter estimates for DSEM models on links between maternal and child behavior.*

| ***Parameter*** | ***Estimate*** | ***95% CI_lower_*** | ***95% CI_upper_*** |
| --- | --- | --- | --- |
| **Interaction Phase: Maternal Social Gaze** |  |  |  |
| **Autoregressive effects (AR)** |  |  |  |
| Maternal Social Gaze à Maternal Social Gaze | **.515** | **.490** | **.539** |
| Child Protest Behavior à Child Protest Behavior | **.656** | **.604** | **.700** |
| Child Self-Regulatory Behavior à Child Self-Regulatory Behavior | **.785** | **.765** | **.805** |
| **Within-Person Cross-lagged effects (CL)** |  |  |  |
| Maternal Social Gaze à Child Protest Behavior | -.004 | -.020 | .010 |
| Maternal Social Gaze à Child Self-Regulatory Behavior | .008 | -.009 | .024 |
|  |  |  |  |
| **Interaction Phase: Maternal Touch** |  |  |  |
| **Autoregressive effects (AR)** |  |  |  |
| Maternal Soothing Touch à Maternal Soothing Touch | **.703** | **.678** | **.728** |
| Child Protest Behavior à Child Protest Behavior | **.654** | **.603** | **.698** |
| Child Self-Regulatory Behavior à Child Self-Regulatory Behavior | **.781** | **.760** | **.801** |
| **Cross-lagged effects (CL)** |  |  |  |
| Maternal Soothing Touch à Child Protest Behavior | .001 | -.019 | .020 |
| Maternal Soothing Touch à Child Self-Regulatory Behavior | .016 | -.001 | .033 |
|  |  |  |  |
| **Interaction Phase: Maternal Positive Vocalizations** |  |  |  |
| **Autoregressive effects (AR)** |  |  |  |
| Maternal Vocalizations à Maternal Vocalizations | **.684** | **.662** | **.705** |
| Child Protest Behavior à Child Protest Behavior | **.648** | **.597** | **.692** |
| Child Self-Regulatory Behavior à Child Self-Regulatory Behavior | **.785** | **.764** | **.804** |
| **Cross-lagged effects (CL)** |  |  |  |
| Maternal Vocalizations à Child Protest Behavior | **-.037** | **-.057** | **-.017** |
| Maternal Vocalizations à Child Self-Regulatory Behavior | -.005 | -.020 | .010 |
|  |  |  |  |
| **Smartphone Phase: Maternal Social Gaze** |  |  |  |
| **Autoregressive effects (AR)** |  |  |  |
| Maternal Social Gaze à Maternal Social Gaze | **.523** | **.495** | **.549** |
| Child Protest Behavior à Child Protest Behavior | **.673** | **.644** | **.700** |
| Child Self-Regulatory Behavior à Child Self-Regulatory Behavior | **.683** | **.647** | **.714** |
| **Within-Person Cross-lagged effects (CL)** |  |  |  |
| Maternal Social Gaze à Child Protest Behavior | -.014 | -.033 | .004 |
| Maternal Social Gaze à Child Self-Regulatory Behavior | .003 | -.014 | .021 |
|  |  |  |  |
| **Smartphone Phase: Maternal Touch** |  |  |  |
| **Autoregressive effects (AR)** |  |  |  |
| Maternal Soothing Touch à Maternal Soothing Touch | **.664** | **.616** | **.705** |
| Child Protest Behavior à Child Protest Behavior | **.674** | **.646** | **.701** |
| Child Self-Regulatory Behavior à Child Self-Regulatory Behavior | **.684** | **.647** | **.715** |
| **Cross-lagged effects (CL)** |  |  |  |
| Maternal Soothing Touch à Child Protest Behavior | -011 | -.034 | .012 |
| Maternal Soothing Touch à Child Self-Regulatory Behavior | .006 | -.016 | .029 |
|  |  |  |  |
| **Smartphone Phase: Maternal Positive Vocalizations** |  |  |  |
| **Autoregressive effects (AR)** |  |  |  |
| Maternal Vocalizations à Maternal Vocalizations | **.703** | **.682** | **.725** |
| Child Protest Behavior à Child Protest Behavior | **.675** | **.646** | **.702** |
| Child Self-Regulatory Behavior à Child Self-Regulatory Behavior | **.682** | **.644** | **.713** |
| **Cross-lagged effects (CL)** |  |  |  |
| Maternal Vocalizations à Child Protest Behavior | -.015 | -.032 | .003 |
| Maternal Vocalizations à Child Self-Regulatory Behavior | .017 | .000 | .034 |
| *Note.* **Bold** indicates significance based on Credible Interval (CI) not containing zero. *All e*stimates are standardized*.* | | | |

Table S4.

*Population level parameter estimates for DSEM models on links between maternal behavior and child cardiac arousal.*

| ***Parameter*** | ***Estimate*** | ***95% CI_lower_*** | ***95% CI_upper_*** |
| --- | --- | --- | --- |
| **Interaction Phase** |  |  |  |
| **Autoregressive effects (AR)** |  |  |  |
| Infant Cardiac Arousal à Infant Cardiac Arousal | **.799** | **.784** | **.814** |
| Maternal Vocalizations à Maternal Vocalizations | **.700** | **.683** | **.716** |
| Maternal Social Gaze à Maternal Social Gaze | **.538** | **.518** | **.558** |
| Maternal Soothing Touch à Maternal Soothing Touch | **.733** | **.717** | **.749** |
| **Within-Person Cross-lagged effects (CL)** |  |  |  |
| Maternal Vocalizations à Infant Cardiac Arousal | .008 | -.008 | .024 |
| Maternal Social Gaze à Infant Cardiac Arousal | -.007 | -.023 | .009 |
| Maternal Soothing Touch à Infant Cardiac Arousal | -.003 | -.019 | .013 |
|  |  |  |  |
| **Smartphone Phase** |  |  |  |
| **Autoregressive effects (AR)** |  |  |  |
| Infant Cardiac Arousal à Infant Cardiac Arousal | **.828** | **.811** | **.844** |
| Maternal Vocalizations à Maternal Vocalizations | **.699** | **.682** | **.717** |
| Maternal Social Gaze à Maternal Social Gaze | **.554** | **.533** | **.574** |
| Maternal Soothing Touch à Maternal Soothing Touch | **.691** | **.672** | **.708** |
| **Within-Person Cross-lagged effects (CL)** |  |  |  |
| Maternal Vocalizations à Infant Cardiac Arousal | -.004 | -.020 | .011 |
| Maternal Social Gaze à Infant Cardiac Arousal | -.008 | -.024 | .007 |
| Maternal Soothing Touch à Infant Cardiac Arousal | -.005 | -.020 | .010 |
|  |  |  |  |
| **Still-Face Phase** |  |  |  |
| **Autoregressive effects (AR)** |  |  |  |
| Infant Cardiac Arousal à Infant Cardiac Arousal | **.853** | **.837** | **.869** |
| Maternal Vocalizations à Maternal Vocalizations | **.631** | **.618** | **.645** |
| Maternal Social Gaze à Maternal Social Gaze | **.617** | **.601** | **.635** |
| Maternal Soothing Touch à Maternal Soothing Touch | **.632** | **.616** | **.647** |
| **Within-Person Cross-lagged effects (CL)** |  |  |  |
| Maternal Vocalizations à Infant Cardiac Arousal | .014 | .000 | .028 |
| Maternal Social Gaze à Infant Cardiac Arousal | -.009 | -.027 | .007 |
| Maternal Soothing Touch à Infant Cardiac Arousal | .009 | -.006 | .024 |
| *Note.* **Bold** indicates significance based on Credible Interval (CI) not containing zero. *All e*stimates are standardized*.* | | | |

Table S5.

*Population level parameter estimates for DSEM models on links between maternal and child cardiac arousal.*

| ***Parameter*** | ***Estimate*** | ***95% CI_lower_*** | ***95% CI_upper_*** |
| --- | --- | --- | --- |
| **Interaction Phase** |  |  |  |
| **Autoregressive effects (AR)** |  |  |  |
| Maternal Cardiac Arousal à Maternal Cardiac Arousal | **.830** | **.815** | **.845** |
| Infant Cardiac Arousal à Infant Cardiac Arousal | **.798** | **.782** | **.814** |
| **Within-Person Cross-lagged effects (CL)** |  |  |  |
| Maternal Cardiac Arousal à Infant Cardiac Arousal | .001 | -.017 | .018 |
| Infant Cardiac Arousal à Maternal Cardiac Arousal | -.009 | -.025 | .007 |
|  |  |  |  |
| **Smartphone Phase** |  |  |  |
| **Autoregressive effects (AR)** |  |  |  |
| Maternal Cardiac Arousal à Maternal Cardiac Arousal | **.835** | **.820** | **.851** |
| Infant Cardiac Arousal à Infant Cardiac Arousal | **.827** | **.810** | **.843** |
| **Within-Person Cross-lagged effects (CL)** |  |  |  |
| Maternal Cardiac Arousal à Infant Cardiac Arousal | -.015 | -.032 | .001 |
| Infant Cardiac Arousal à Maternal Cardiac Arousal | **-.029** | **-.045** | **-.013** |
|  |  |  |  |
| **Still-Face Phase** |  |  |  |
| **Autoregressive effects (AR)** |  |  |  |
| Maternal Cardiac Arousal à Maternal Cardiac Arousal | **.864** | **.850** | **.877** |
| Infant Cardiac Arousal à Infant Cardiac Arousal | **.849** | **.833** | **.865** |
| **Within-Person Cross-lagged effects (CL)** |  |  |  |
| Maternal Cardiac Arousal à Infant Cardiac Arousal | **-.021** | **-.037** | **-.005** |
| Infant Cardiac Arousal à Maternal Cardiac Arousal | **-.033** | **-.047** | **-.019** |
| *Note.* **Bold** indicates significance based on Credible Interval (CI) not containing zero. *All e*stimates are standardized*.* | | | |
